# Construction and validation of a frailty screening model for older adults with cataract: a cross-sectional study

**DOI:** 10.3389/fmed.2026.1934999

**Published:** 2026-10-01

**Authors:** Xingdong Cheng, Heting Liang, Qingyun Mao, Yumeng Zhang, Shaoting Yang, Honghong Wen, Xiaoli Yuan

**Affiliations:** 1 Department of Nursing, Affiliated Hospital of Zunyi Medical University, Zunyi, Guizhou, China; 2 Faculty of Nursing, Zunyi Medical University, Zunyi, Guizhou, China

**Keywords:** associated factors, cataract, frailty, older adults, risk assessment model

## Abstract

**Objective:**

To develop and internally validate a frailty screening model for older adults with cataract that relies only on information already collected during routine ophthalmology outpatient assessment.

**Methods:**

In this cross-sectional study, 516 patients aged ≥ 60 years were enrolled and randomly divided into training (n = 361) and internal validation (*n* = 155) sets. Frailty was defined by the FRAIL scale, with non-frail and pre-frail categories combined for the primary binary analysis. Univariate screening was followed by multivariable binary logistic regression, with LASSO and a 5,000-resample bootstrap of the full development procedure as stability checks.

**Results:**

The frailty detection rate was 29.07%. Five factors were independently associated with frailty: advanced age, IADL impairment, poor self-rated health, depression, and history of falls. Discrimination was good (training AUC 0.841, 95% CI 0.796–0.887; validation AUC 0.816, 95% CI 0.745–0.888), the optimism-corrected C-statistic was 0.810 (95% CI 0.780–0.841), and calibration was acceptable (validation Brier score 0.1553). Decision curve analysis showed positive net benefit across a broad threshold range. Lowering the operating point from the Youden cutoff (0.463; sensitivity 57.8%, specificity 86.4%) to 0.181 raised validation sensitivity to 86.7% with a negative predictive value of 92.1%.

**Conclusions:**

Because all five predictors are already recorded during routine cataract assessment, the model offers an opportunistic, zero-burden triage step for identifying concurrent frailty and prompting comprehensive geriatric assessment. External validation and comparison with the FRAIL scale are required.

## 1 Introduction

Frailty is a geriatric syndrome characterized by age-related decline in physiological reserves and intrinsic capacity, and is associated with significantly increased vulnerability to stressors and heightened risk of various adverse health outcomes (1–3). Globally, the prevalence of frailty ranges from 12% to 24%, making it a widespread public health concern (4, 5). In China, the pooled prevalence of frailty among community-dwelling Chinese older adults aged 60 years and above has been reported at approximately 10% in a recent meta-analysis and rises markedly with age (6). Frailty is associated with progressive functional decline, which may impair the ability to maintain independent living and engage in social interactions. Studies indicate that, compared to healthy older adults, those with frailty are more susceptible to cognitive impairment, which in turn increases the risk of adverse outcomes. These consequences not only significantly diminish quality of life but also elevate the incidence of unplanned hospital admissions, placing a substantial burden on healthcare resources (4, 5).

Cataract is the leading cause of blindness worldwide (7), with a global prevalence of 17.2%. In China, the prevalence reaches as high as 22.78%, and among the population aged 80 and above, it approaches nearly 100% (8, 9). Characterized by painless and progressive vision loss, the condition often progresses subtly and is frequently unnoticed in its early stages. This issue is particularly pronounced in economically underdeveloped regions, where regular eye disease screening is generally lacking among residents (7). Vision impairment caused by cataracts significantly limits individuals' ability to perform daily activities, reduces social participation, and increases the risk of falls, and is associated with the presence of frailty (10, 11). In this context, cataract may act not merely as a visual problem but also as a factor associated with the increased risk of falls and fractures in older adults (10, 12). Evidence suggests that approximately every six cataract surgeries can prevent one fracture (12). Since both cataracts and frailty are age-related, they often coexist. A study revealed that the prevalence of frailty among Chinese cataract patients is as high as 58.88% (13). Another four-year follow-up study of Chinese older adults found that the risk of developing frailty among those with visual impairment was approximately twice as high as among those without (14). On the other hand, the state of frailty reduces patients' willingness and opportunities to undergo surgery, further exacerbating vision loss (15).

According to the World Health Organization (WHO), globally only a minority of people with cataract-related vision impairment or blindness receive surgical treatment (7). In practice, the cataract clinic is a natural and low-cost site for frailty detection. These patients are already presenting for eye care, so the same visit can be used to screen for frailty and, if positive, to prompt a comprehensive geriatric work-up. To our knowledge, dedicated frailty screening tools for older adults with cataract remain limited, making accurate screening for prevalent frailty and subsequent stratified management difficult. Therefore, rather than modelling cataract-specific pathophysiology, this study aimed to develop and internally validate, within this readily accessible care setting, a frailty screening tool that integrates multidimensional variables (sociodemographic, functional, psychological, and health-related) to explore the associated factors of frailty in older adults with cataract. Although the FRAIL scale is a widely used screening tool (16, 17), it may not fully capture the multifactorial risk profile of this population; a transparent, setting-embedded nomogram may help clinicians identify patients at high probability of concurrent frailty earlier and direct them to appropriate geriatric or multidisciplinary evaluation.

## 2 Material and methods

### 2.1 Study population

A convenience sampling method was used in this study. Cataract patients who attended the ophthalmology outpatient clinic of a tertiary hospital in Zunyi City, China, from October 2022 to September 2023 were recruited in this study. The inclusion criteria were as follows: (1) age ≥ 60 years; (2) ability to communicate effectively; (3) voluntary participation in the study and sign the consent form. The exclusion criteria were: (1) patients with severe mental illnesses; (2) critically ill or terminally ill patients. Ethics approval for the study was granted by the Ethics Committee of the Affiliated Hospital of Zunyi Medical University (KLL2022-814). The study was conducted in accordance with the principles outlined in the Declaration of Helsinki, and all the participants signed the consent form at the time of participation. The screening and exclusion process is summarised in Figure 1: of 564 cataract outpatients screened, 48 were excluded (2 with severe psychiatric or cognitive disorders, 29 who declined participation or had missing informed consent, and 17 with incomplete questionnaire data), leaving 516 patients who constituted the analytic sample.

**Figure 1:**
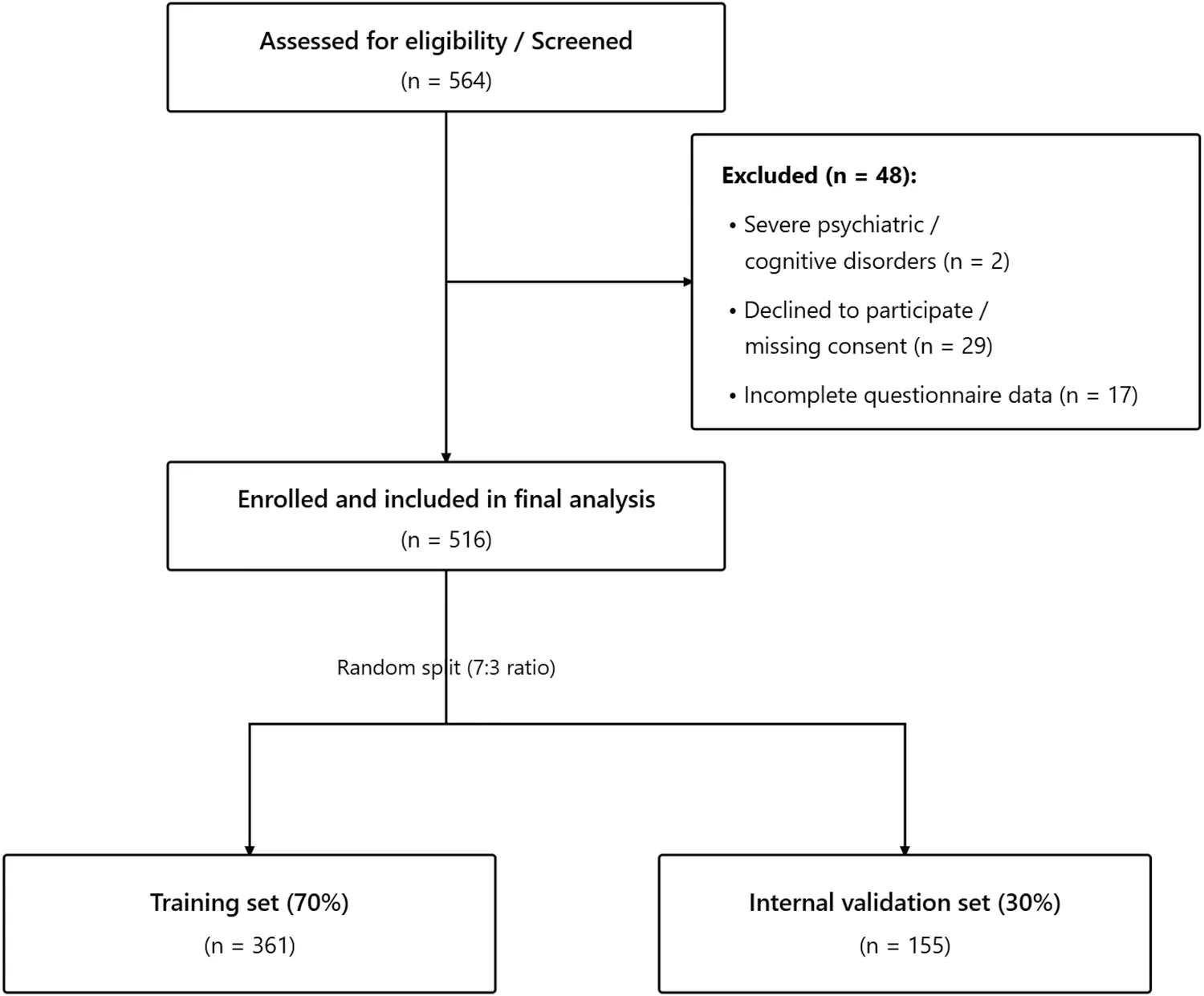
Participant flow diagram. A total of 564 older cataract outpatients were screened, 48 were excluded, and 516 were enrolled and included in the final analysis; the analytic sample was divided into a training set (*n* = 361) and an internal validation set (*n* = 155) by a 7:3 stratified random split.

The sample size was estimated using the 10 events per variable (EPV) method as an a-priori lower bound. This study aimed to develop a model to screen for frailty in older adults with cataract, with frailty status as the outcome variable. The conventional 10 EPV rule was applied to the expected size of the FINAL multivariable model (≤10 predictors), and an external event rate of 58.88% from a single-centre Chinese study (13) was used, giving a training-set minimum of 170 participants (with a 0.7 training proportion). Accounting for ∼20% invalid or incomplete samples, the total minimum sample required was 292 participants. Because the study actually considered 22 candidate predictors, several with three or four categories, the degrees of freedom consumed during model development (including the univariable pre-screening step) were larger than the final model size; the achieved *n* = 516 is therefore presented. A *post-hoc* sample-size check based on the observed event rate (29.07%) and 5–10 candidate parameters is reported in Supplementary Table S2. The sample size (516) of this study is sufficient.

### 2.2 Assessment tools

#### 2.2.1 Sociodemographic characteristics and health status

Self-designed by researchers, it includes gender, age, educational level, marital status, residence type, living arrangement, medical insurance, per capita monthly household income (yuan), comorbid chronic diseases, alcohol consumption, smoking status, annual physical examination, social activity participation, physical exercise, social support, self-reported hearing status and self-rated health status. No cataract-specific clinical variables (objective visual acuity, cataract type, cataract severity, or laterality) were collected, so the variables available for modelling were limited to general geriatric information that is already recorded during routine cataract assessment.

#### 2.2.2 Instrumental activities of daily living (IADL)

IADL was measured by Lawton Instrumental Activities of Daily Living Scale, which developed by Lawton et al. (18), the scale comprises 8 items: using the telephone, shopping, meal preparation, housekeeping, laundry, transportation, medication management, and financial management. Each item is scored 1 (independence) or 0 (dependence), with a total score ranging from 0 to 8. A full score indicates independent ability; an IADL total score < 8 was defined as IADL impairment. In this study, the Cronbach's *α* was 0.908.

#### 2.2.3 Depression

Depression was measured by Short Form Geriatric Depression Scale (GDS-15), which derived from the 30-item original version by Sheikh and Yesavage (19) in 1986, this abbreviated scale has been widely used internationally. Its Chinese version demonstrated good reliability and validity among urban and rural elderly in China, as validated by Tang et al. (20) in 2013, making it suitable for assessing depressive symptoms in Chinese older adults. The 15-item scale uses “Yes/No” responses, scored 1/0 respectively, with total scores ranging 0-15: 0-4 (normal), 5-9 (mild depression), ≥10 (severe depression). In this study, the Cronbach's α was 0.788.

#### 2.2.4 Anxiety

Anxiety was measured by Self-Rating Anxiety Scale (SAS), which developed by Prof. W.K. Zung (21), the SAS consists of 20 items scored 1–4. The raw score (sum of items) is multiplied by 1.25 and rounded to an integer. A total score <50 indicates normal anxiety; 50–59, mild anxiety; 60–69, moderate anxiety; and ≥70, severe anxiety. In this study, the Cronbach's *α* was 0.841.

#### 2.2.5 Sleep quality

Sleep quality was measured by Pittsburgh Sleep Quality Index (PSQI), which developed by Dr. Buysse et al. from the University of Pittsburgh in 1989 (22), the PSQI comprises 18 items grouped into 7 components. The total score (sum of components) ranges 0–21: 0–5 indicates very good sleep quality; 6–10, okay; 11–15, poor; and 16–21, very poor. In this study, the Cronbach's *α* was 0.765.

#### 2.2.6 History of falls

History of falls was assessed by asking patients: “Have you fallen in the past 12 months?” Patients who responded “Yes” were classified as having a history of falls, while those who responded “No” were classified as not having a history of falls.

#### 2.2.7 Frailty

Frailty was measured by the FRAIL scale, which was recommended in Chinese experts consensus on assessment and intervention for elderly patients with frailty (16) for assessing geriatric frailty, this scale includes 5 items: fatigue (frequent tiredness), stair climbing (ability to climb 1 flight unassisted), walking (ability to walk 100 meters unassisted), comorbidities (≥5 chronic diseases), and weight loss (≥5% body weight loss in the past year). Each item scores 1 point (total 0–5). Frailty is classified as: non-frailty (0), pre-frailty (1–2), and frailty (≥3). In this study, participants were categorized into a non-frailty group (including both non-frailty and pre-frailty) and a frailty group. The Cronbach's *α* of this study was 0.711.

### 2.3 Data analysis

All statistical analyses were performed using Python 3.13 (pandas, statsmodels, and matplotlib), and model development followed the TRIPOD reporting framework (23). Categorical variables were compared between groups using the χ² test or Fisher's exact test.

Univariate analysis was conducted on the training set only; variables with *P* < 0.05 were entered as candidates into a multivariable binary logistic regression model; after mutual adjustment, only variables with an independently significant association (*P* < 0.05) were retained, yielding the final parsimonious model (24). This sequential procedure avoids using the validation set for variable selection or model estimation. To address the well-known instability of significance-based pre-screening, a prespecified secondary model was fit using L1-penalised logistic regression (LASSO) on all 22 candidate predictors with the regularization parameter C tuned by stratified 5-fold cross-validation of the negative log-likelihood. For internal validation, the bootstrap procedure repeated the complete model-development pipeline (univariable screening, multivariable selection, and refitting of the reduced model) in each of 5,000 resamples, and the resample-trained model was evaluated on the original training set; the optimism-corrected C-statistic and its 2.5th–97.5th percentile confidence interval are reported. The detailed training-set univariate results are reported in Supplementary Table S1. In the present dataset, all independent variables had variance inflation factor (VIF) < 10 and tolerance > 0.1, indicating no significant multicollinearity.

A nomogram was constructed in Python based on the variables retained in the final multivariable model. Discriminative ability was evaluated by ROC analysis and the AUC. Calibration was assessed by a decile-based calibration plot, the Hosmer–Lemeshow test, the mean absolute error (MAE), the calibration intercept and slope from logistic recalibration, and the Brier score. Robust internal validation was performed by 5,000 bootstrap resamples with the full model-development pipeline repeated in each resample; the optimism-corrected C-statistic was obtained by subtracting the mean optimism across resamples from the apparent C-statistic. The incremental discriminative value of the full model over (i) an age-only baseline and (ii) an age + sex + comorbid baseline was assessed in the validation set using the DeLong test, the continuous net reclassification improvement (NRI), and the integrated discrimination improvement (IDI); the Brier score of each comparator is also reported. To assess clinical utility, decision curve analysis (DCA) was performed over the threshold range 0.01–0.99 and the threshold range yielding positive net benefit was reported. Because the Youden-index sensitivity of 57.8% in the validation set is below commonly recommended screening thresholds, we additionally report the specificity, positive predictive value, and negative predictive value at sensitivity targets of 80%, 85%, and 90% to support threshold selection for a screening context. A three-level outcome sensitivity analysis was performed by fitting an ordinal logistic regression model on the original (non-frail/pre-frail/frail) trichotomy; the proportional-odds assumption was tested by a likelihood-ratio comparison with a multinomial model. An additional sensitivity analysis was conducted by replacing age categories with restricted cubic splines (3 knots at the 10th, 50th, and 90th percentiles). A two-tailed *P* < 0.05 was considered statistically significant.

## 3 Results

### 3.1 Description of sociodemographic characteristics and health status

This study included a total of 516 participants, among whom 150 (29.07%) were identified as frail (FRAIL scale score ≥ 3), 172 (33.33%) as pre-frail (score 1–2), and 194 (37.60%) as non-frail (score 0). For the primary analysis, the non-frail and pre-frail categories were combined into a single non-frail group to support binary logistic regression; the original three-level structure was retained for an ordinal-logistic sensitivity analysis (Supplementary Table S2).

The majority of participants were female (63.37%). Age ranged from 60 to 98 years, with the largest proportion in the 70–79 age group (49.42%). In the non-frail group, the most common age range was 60–69 years (31.97%), whereas in the frail group, individuals aged 80 years or older accounted for a higher proportion (35.33%). The overall educational level was low, with 73.06% having an education level of primary school or below. The majority of participants were married (70.35%) and lived with others (91.28%). Additionally, 60.66% reported a monthly per capita household income below 2,000 Yuan.

Health insurance coverage reached 98.26%. The prevalence of chronic diseases was higher in the frail group (88.67%) compared to the non-frail group (71.86%). Most participants were non-drinkers (91.67%) and non-smokers (87.02%). The non-frail group had higher proportions of annual physical examinations (64.75% vs. 52.00%), regular physical exercise (82.24% vs. 55.33%), and participation in social activities (47.81% vs. 29.33%) compared to the frail group.

The frail group exhibited significantly higher proportions of IADL impairment (91.33% vs. 62.30%) and history of falls within the past 12 months (36.67% vs. 16.39%) compared to the non-frail group. The frail group had higher proportions of Severe depression and mild-to-moderate anxiety. In terms of sleep quality, the non-frail group reported a higher proportion of “very good” sleep quality (19.95% vs. 10.00%), while the frail group had higher proportions of “poor” and “very poor” sleep quality. Among the demographic and health-related characteristics, age, type of residence, comorbid chronic diseases, annual physical examination, social activity participation, physical exercise, IADL impairment, self-rated health status, depression, anxiety, sleep quality, and history of falls showed statistically significant differences between the non-frail and frail groups (*P* < 0.05), Table 1.

**Table 1:** Sociodemographic characteristics and health status of the participants.

Variables	Total (*n* = 516)	Non-frail/pre-frail group (*n* = 366)	Frail group (*n* = 150)	χ²	*P*
Gender				1.486^a^	0.223
Male	189 (36.63)	128 (34.97)	61 (40.67)		
Female	327 (63.37)	238 (65.03)	89 (59.33)		
Age				23.586^a^	<0.001
60–69 years	140 (27.13)	117 (31.97)	23 (15.33)		
70–79 years	255 (49.42)	181 (49.45)	74 (49.33)		
≥80 years	121 (23.45)	68 (18.58)	53 (35.33)		
Educational level				1.229^a^	0.541
Primary school or below	377 (73.06)	272 (74.32)	105 (70.00)		
Junior high school	78 (15.12)	54 (14.75)	24 (16.00)		
Senior high school or above	61 (11.82)	40 (10.93)	21 (14.00)		
Marital status				2.550^a^	0.110
Married	363 (70.35)	265 (72.40)	98 (65.33)		
Unmarried	153 (29.65)	101 (27.60)	52 (34.67)		
Residence type				4.532^a^	0.033
Urban	292 (56.59)	218 (59.56)	74 (49.33)		
Rural	224 (43.41)	148 (40.44)	76 (50.67)		
Living arrangement				0.100^a^	0.752
Living with others	471 (91.28)	335 (91.53)	136 (90.67)		
Living alone	45 (8.72)	31 (8.47)	14 (9.33)		
Medical insurance				0.007^a^	0.931
Yes	507 (98.26)	359 (98.09)	148 (98.67)		
No	9 (1.74)	7 (1.91)	2 (1.33)		
Per capita monthly household income (1 USD ≈ 7.079 Yuan)				0.357^a^	0.550
≥2,000	203 (39.34)	147 (40.16)	56 (37.33)		
<2,000	313 (60.66)	219 (59.84)	94 (62.67)		
Comorbid chronic diseases				16.843^a^	<0.001
No	120 (23.26)	103 (28.14)	17 (11.33)		
Yes	396 (76.74)	263 (71.86)	133 (88.67)		
Alcohol consumption				3.722^a^	0.054
Non-drinker	473 (91.67)	330 (90.16)	143 (95.33)		
Drinker	43 (8.33)	36 (9.84)	7 (4.67)		
Smoking status				0.510^a^	0.475
Non-smoker	449 (87.02)	316 (86.34)	133 (88.67)		
Smoker	67 (12.98)	50 (13.66)	17 (11.33)		
Annual physical examination				7.278^a^	0.007
Yes	315 (61.05)	237 (64.75)	78 (52.00)		
No	201 (38.95)	129 (35.25)	72 (48.00)		
Social activity participation				14.875^a^	<0.001
Yes	219 (42.44)	175 (47.81)	44 (29.33)		
No	297 (57.56)	191 (52.19)	106 (70.67)		
Physical exercise				40.462^a^	<0.001
Yes	384 (74.42)	301 (82.24)	83 (55.33)		
No	132 (25.58)	65 (17.76)	67 (44.67)		
Social support				0.730^a^	0.393
Yes	462 (89.53)	325 (88.80)	137 (91.33)		
No	54 (10.47)	41 (11.20)	13 (8.67)		
Hearing impairment				2.433^a^	0.119
No	411 (79.65)	298 (81.42)	113 (75.33)		
Yes	105 (20.35)	68 (18.58)	37 (24.67)		
IADL impairment				43.340^a^	<0.001
No	151 (29.26)	138 (37.70)	13 (8.67)		
Yes	365 (70.74)	228 (62.30)	137 (91.33)		
Self-rated health status				79.657^a^	<0.001
Good	83 (16.09)	77 (21.04)	6 (4.00)		
Fair	322 (62.40)	246 (67.21)	76 (50.67)		
Poor	111 (21.51)	43 (11.75)	68 (45.33)		
Depression				58.437^a^	<0.001
Normal	349 (67.64)	284 (77.60)	65 (43.33)		
Mild depression	135 (26.16)	69 (18.85)	66 (44.00)		
Severe depression	32 (6.20)	13 (3.55)	19 (12.67)		
Anxiety				9.717^b^	0.013
Normal	409 (79.26)	300 (81.97)	109 (72.67)		
Mild anxiety	89 (17.25)	58 (15.85)	31 (20.67)		
Moderate anxiety	17 (3.29)	7 (1.91)	10 (6.67)		
Severe anxiety	1 (0.19)	1 (0.27)	0 (0.00)		
Sleep Quality				18.186^a^	<0.001
Very good	88 (17.05)	73 (19.95)	15 (10.00)		
Okay	244 (47.29)	181 (49.45)	63 (42.00)		
Fair	167 (32.36)	104 (28.42)	63 (42.00)		
Very poor	17 (3.29)	8 (2.19)	9 (6.00)		
History of falls				25.248^a^	<0.001
No	401 (77.71)	306 (83.61)	95 (63.33)		
Yes	115 (22.29)	60 (16.39)	55 (36.67)		

a χ² test.

b Fisher exact test.

### 3.2 Logistic regression analysis of frailty in older adults with cataract

Variables that showed statistical significance in the univariate analysis performed on the training set were entered into a multivariate binary logistic regression analysis. The results indicated that advanced age [with 60–69 years as reference: 70–79 years: OR (95% CI) = 1.564 (0.745–3.280), *P* = 0.237; ≥80 years: OR (95% CI) = 2.880 (1.260–6.583), *P* = 0.012], IADL impairment [with IADL independence as reference: OR (95% CI) = 3.896 (1.684–9.012), *P* = 0.001], self-rated health status [with good as reference: fair: OR (95% CI) = 4.385 (1.432–13.429), *P* = 0.010; poor: OR (95% CI) = 19.030 (5.820–62.223), *P* < 0.001], depression [with normal as reference: mild depression: OR (95% CI) = 3.117 (1.712–5.675), *P* < 0.001; severe depression: OR (95% CI) = 3.781 (1.097–13.025), *P* = 0.035], and history of falls [with no history of falls as reference: OR (95% CI) = 2.025 (1.080–3.796), *P* = 0.028] were factors significantly associated with frailty (*P* < 0.05; Table 2). Two estimates had wide confidence intervals reflecting sparse cells: self-rated health ‘poor’ (events in the poor-self-rated-health  ×  frail stratum were limited) and severe depression (*n* = 32 in the full sample, of whom 19 were frail). These estimates should be interpreted with caution; collapsing the severe-depression cell with mild depression (post-hoc) or re-estimating the model after excluding the sparse severe-anxiety stratum (*n* = 1) did not materially change the AUC (0.841 vs. 0.841) or the retained predictors. As a complementary stability check, a LASSO logistic regression on all 22 prespecified predictors retained 17 non-zero coefficients and yielded a near-identical validation AUC (0.812), supporting the overall ranking of predictors.

**Table 2:** Logistic regression analysis of frailty in older adults with cataract.

Variables	β	S.E	Z	*P*	OR (95%CI)
Intercept	−4.830	0.720	−6.708	<0.001	0.008 (0.002–0.033)
Age					
60–69 years					1.000 (Reference)
70–79 years	0.447	0.378	1.182	0.237	1.563 (0.745–3.280)
≥80 years	1.058	0.422	2.507	0.012	2.880 (1.260–6.583)
IADL impairment					
No					1.000 (Reference)
Yes	1.360	0.428	3.179	0.001	3.896 (1.684–9.012)
Self-rated health status					
Good					1.000 (Reference)
Fair	1.478	0.571	2.588	0.010	4.385 (1.432–13.429)
Poor	2.946	0.604	4.874	<0.001	19.030 (5.820–62.223)
Depression					
Normal					1.000 (Reference)
Mild depression	1.137	0.306	3.715	<0.001	3.117 (1.712–5.675)
Severe depression	1.330	0.630	2.111	0.035	3.781 (1.097–13.025)
History of falls					
No					1.000 (Reference)
Yes	0.705	0.320	2.203	0.028	2.025 (1.080–3.796)

### 3.3 Construction of a frailty screening nomogram for older adults with cataract

The dataset was randomly divided into a training set (*n* = 361) and a validation set (*n* = 155) at a ratio of 7:3 (seed = 345), stratified by frailty status. No statistically significant differences were observed between the two sets in baseline characteristics (all *P* > 0.05), as shown in Table 3. Using the training set data, a nomogram was constructed based on the results of the multivariate logistic regression analysis. The final model incorporated the following factors: age, IADL, self-rated health status, depression, and history of falls, as shown in Figure 2.

**Table 3:** Comparison of the training set and the validation set.

Variables	Total (*n* = 516)	Training set (*n* = 361)	Validation set (*n* = 155)	χ²	*P*
Gender				0.710^a^	0.400
Male	189 (36.63)	128 (35.46)	61 (39.35)		
Female	327 (63.37)	233 (64.54)	94 (60.65)		
Age				2.320^a^	0.313
60–69 years	140 (27.13)	91 (25.21)	49 (31.61)		
70–79 years	255 (49.42)	182 (50.42)	73 (47.10)		
≥80 years	121 (23.45)	88 (24.38)	33 (21.29)		
Educational level				2.450^a^	0.294
Primary school or below	377 (73.06)	267 (73.96)	110 (70.97)		
Junior high school	78 (15.12)	49 (13.57)	29 (18.71)		
Senior high school or above	61 (11.82)	45 (12.47)	16 (10.32)		
Marital status				0.180^a^	0.668
Married	363 (70.35)	256 (70.91)	107 (69.03)		
Unmarried	153 (29.65)	105 (29.09)	48 (30.97)		
Residence type				0.110^a^	0.740
Urban	292 (56.59)	206 (57.06)	86 (55.48)		
Rural	224 (43.41)	155 (42.94)	69 (44.52)		
Living arrangement				3.480^a^	0.062
Living with others	471 (91.28)	335 (92.80)	136 (87.74)		
Living alone	45 (8.72)	26 (7.20)	19 (12.26)		
Medical insurance				0.020^a^	0.881
Yes	507 (98.26)	354 (98.06)	153 (98.71)		
No	9 (1.74)	7 (1.94)	2 (1.29)		
Per capita monthly household income (Yuan) (1 USD ≈ 7.079 Yuan)				0.340^a^	0.558
≥2,000	203 (39.34)	145 (40.17)	58 (37.42)		
<2,000	313 (60.66)	216 (59.83)	97 (62.58)		
Comorbid chronic diseases				0.200^a^	0.657
No	120 (23.26)	82 (22.71)	38 (24.52)		
Yes	396 (76.74)	279 (77.29)	117 (75.48)		
Alcohol consumption				0.001^a^	0.977
Non-drinker	473 (91.67)	331 (91.69)	142 (91.61)		
Drinker	43 (8.33)	30 (8.31)	13 (8.39)		
Smoking status				1.940^a^	0.164
Non-smoker	449 (87.02)	319 (88.37)	130 (83.87)		
Smoker	67 (12.98)	42 (11.63)	25 (16.13)		
Annual physical examination				0.740^a^	0.389
Yes	315 (61.05)	216 (59.83)	99 (63.87)		
No	201 (38.95)	145 (40.17)	56 (36.13)		
Social activity participation				0.860^a^	0.353
Yes	219 (42.44)	158 (43.77)	61 (39.35)		
No	297 (57.56)	203 (56.23)	94 (60.65)		
Physical exercise				0.540^a^	0.461
Yes	384 (74.42)	272 (75.35)	112 (72.26)		
No	132 (25.58)	89 (24.65)	43 (27.74)		
Social support				1.750^a^	0.185
Yes	462 (89.53)	319 (88.37)	143 (92.26)		
No	54 (10.47)	42 (11.63)	12 (7.74)		
Hearing impairment				1.130^a^	0.287
No	411 (79.65)	292 (80.89)	119 (76.77)		
Yes	105 (20.35)	69 (19.11)	36 (23.23)		
IADL impairment				0.080^a^	0.774
No	151 (29.26)	107 (29.64)	44 (28.39)		
Yes	365 (70.74)	254 (70.36)	111 (71.61)		
Self-rated health status				1.780^a^	0.410
Good	83 (16.09)	55 (15.24)	28 (18.06)		
Fair	322 (62.40)	232 (64.27)	90 (58.06)		
Poor	111 (21.51)	74 (20.50)	37 (23.87)		
Depression				1.110^a^	0.575
Normal	349 (67.64)	243 (67.31)	106 (68.39)		
Mild depression	135 (26.16)	93 (25.76)	42 (27.10)		
Severe depression	32 (6.20)	25 (6.93)	7 (4.52)		
Anxiety				1.579^b^	0.736
Normal	409 (79.26)	284 (78.67)	125 (80.65)		
Mild anxiety	89 (17.25)	62 (17.17)	27 (17.42)		
Moderate anxiety	17 (3.29)	14 (3.88)	3 (1.94)		
Severe anxiety	1 (0.19)	1 (0.28)	0 (0.00)		
Sleep Quality				0.170^a^	0.982
Very good	88 (17.05)	60 (16.62)	28 (18.06)		
Okay	244 (47.29)	172 (47.65)	72 (46.45)		
Fair	167 (32.36)	117 (32.41)	50 (32.26)		
Very poor	17 (3.29)	12 (3.32)	5 (3.23)		
History of falls				1.100^a^	0.294
No	401 (77.71)	276 (76.45)	125 (80.65)		
Yes	115 (22.29)	85 (23.55)	30 (19.35)		
Frailty				0.040^a^	0.842
Non-frail group	366 (70.93)	257 (71.19)	109 (70.32)		
Frail group	150 (29.07)	104 (28.81)	46 (29.68)		

a χ² test.

b Fisher exact test.

**Figure 2:**
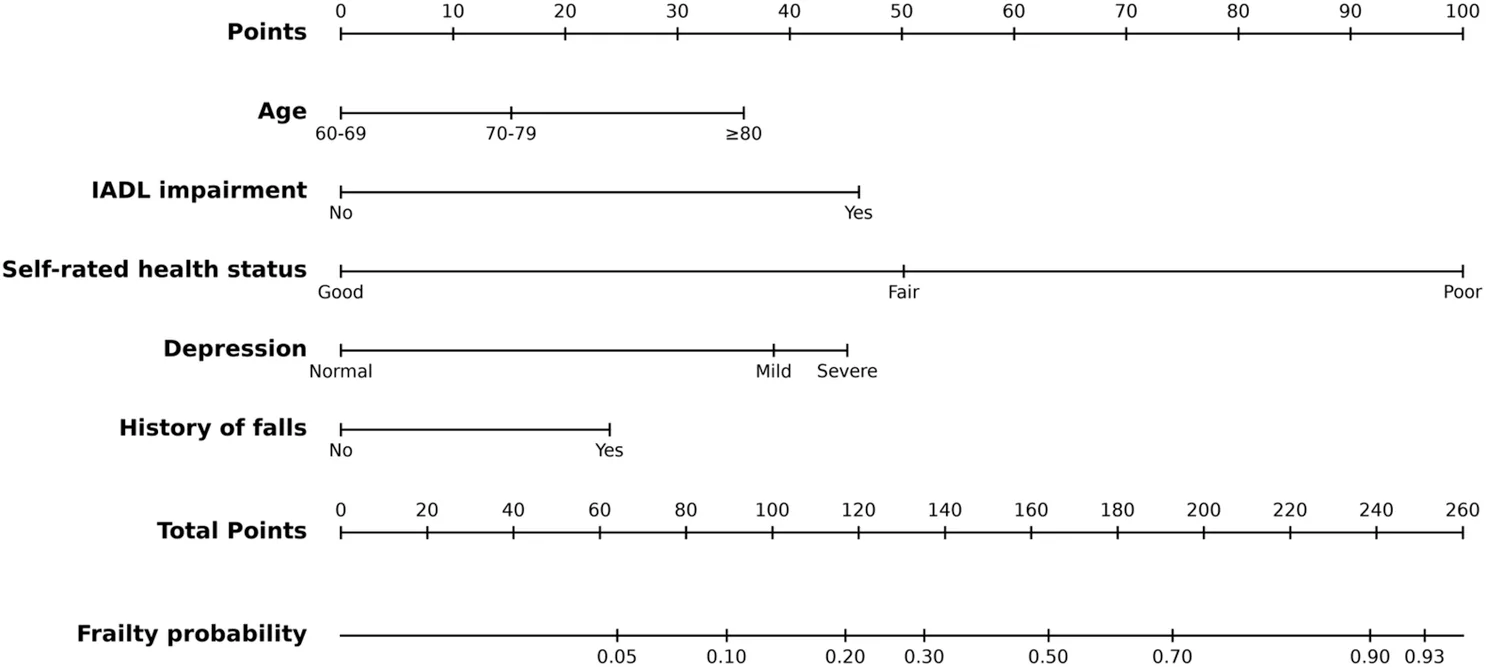
Nomogram for frailty screening in older adults with cataract.

### 3.4 Validation of the frailty screening model in older adults with cataract

Receiver operating characteristic (ROC) curves were plotted for both the training and validation sets, and the AUC was calculated. As shown in Figure 3, the AUC was 0.841 (95% CI: 0.796–0.887) for the training set and 0.816 (95% CI: 0.745–0.888) for the validation set, indicating good discriminative ability of the model. Bootstrap internal validation with the full model-development procedure repeated in each of 5,000 resamples yielded an optimism-corrected C-statistic of 0.810 (95% CI: 0.780–0.841). The optimal cutoff value was determined in the training set using the Youden index to be 0.463, with corresponding sensitivity and specificity of 64.80% and 90.20% in the training set and 57.80% and 86.40% in the validation set, respectively.

**Figure 3:**
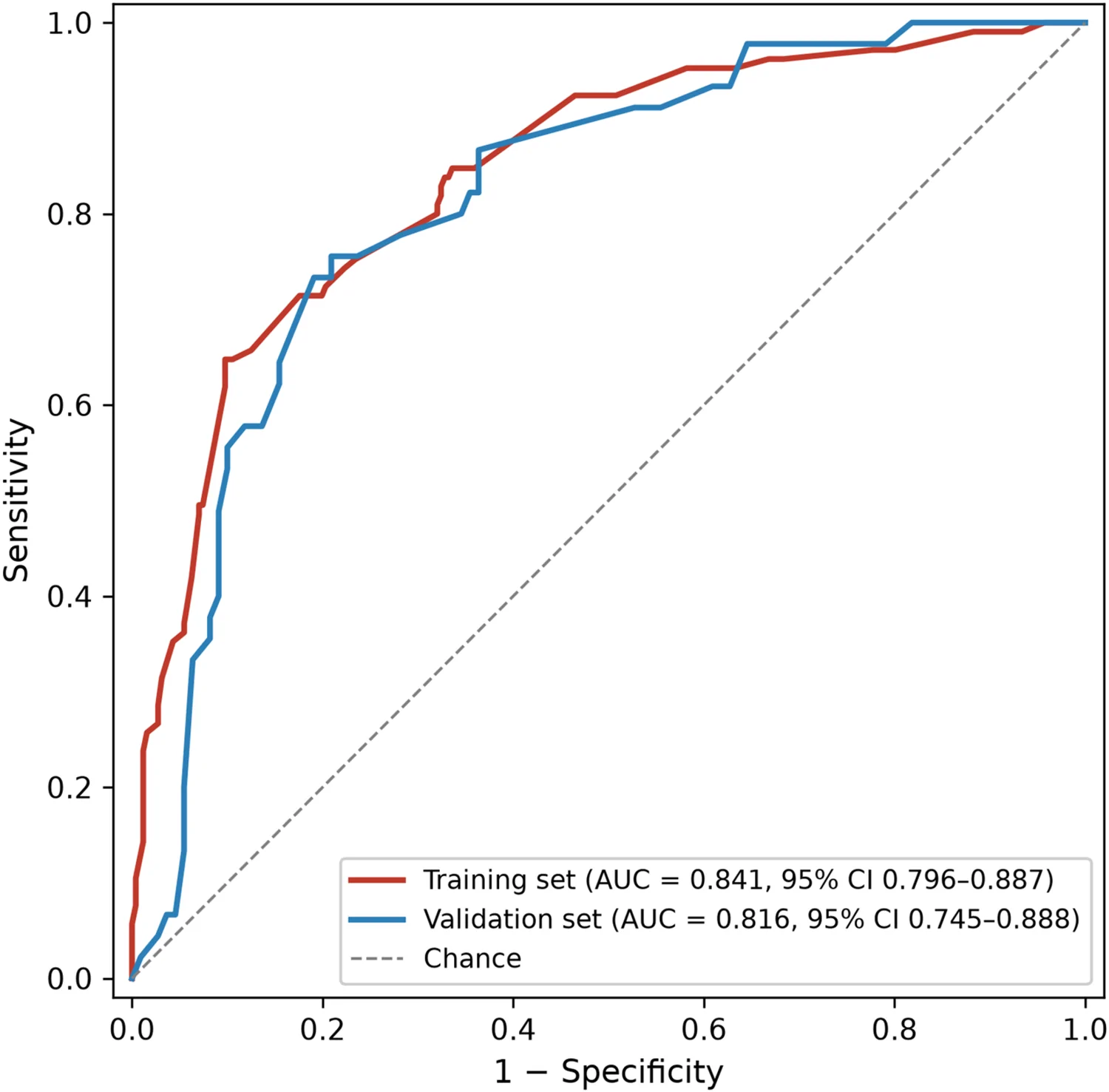
ROC curves of the frailty screening model in the training and validation sets. ROC, receiver operating characteristic; AUC, area under the curve.

Calibration curves for the training set (Figure 4A) and validation set (Figure 4B) showed mean absolute errors (MAE) of 0.051 and 0.075, respectively, and the Hosmer–Lemeshow test results were *χ*² = 9.741 (*P* = 0.284, training) and *χ*² = 7.268 (*P* = 0.508, validation). Because a non-significant Hosmer–Lemeshow test does not exclude miscalibration in a dataset of this size, additional calibration measures are reported. Logistic recalibration intercepts were −2.80 (training) and −2.54 (validation) and calibration slopes were 5.64 (training) and 4.73 (validation); slopes above 1 indicate that the spread of the predicted probabilities was conservative relative to the observed event proportions, so that external recalibration (intercept and slope adjustment) would further refine probability estimation in more diverse populations. The Brier scores were 0.1395 (training) and 0.1553 (validation), below the null-model Brier of 0.206 (event rate 29.07%). Decile-group calibration with a superimposed LOWESS smoother is shown for both sets in Figure 4. Decision curve analysis (Figure 5) showed positive net benefit over both the treat-all and treat-none strategies in the threshold range 0.01–0.92 (training) and 0.01–0.65 (validation). The full model demonstrated a significantly higher AUC than the age-only baseline in the validation set (0.816 vs. 0.636; DeLong *P* < 0.001) and than a stronger age + sex + comorbid baseline (0.816 vs. 0.708; DeLong *P* = 0.026); the continuous NRI was 0.383, the IDI was 0.1897, and the Brier score of the full model (0.1553) was lower than that of the age + sex + comorbid model (0.1858), supporting the incremental value of integrating functional, psychological, and self-rated-health information beyond basic demographic and comorbidity information. At the conventional Youden cutoff (0.463, determined in the training set) the validation-set sensitivity was 57.8% with a specificity of 86.4%. Because the Youden index is a purely statistical balance point rather than a clinically mandated threshold, the operating points at sensitivity targets of 80%, 85%, and 90% are reported in Supplementary Table S2 and Figure 6 so that the threshold can be set according to the intended use.

**Figure 4:**
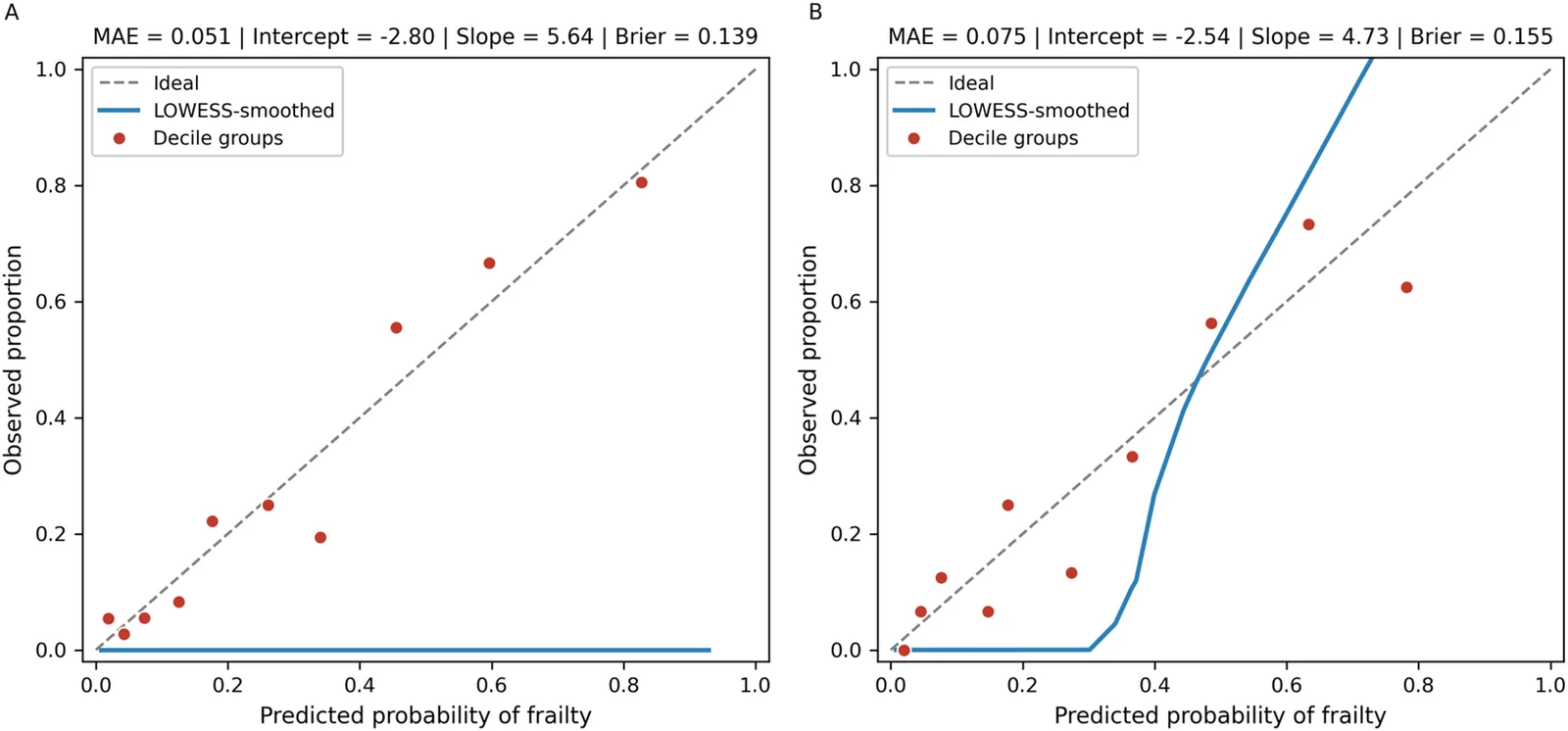
Calibration plots for the frailty screening model. **(A)** Training set. **(B)** Validation set.

**Figure 5:**
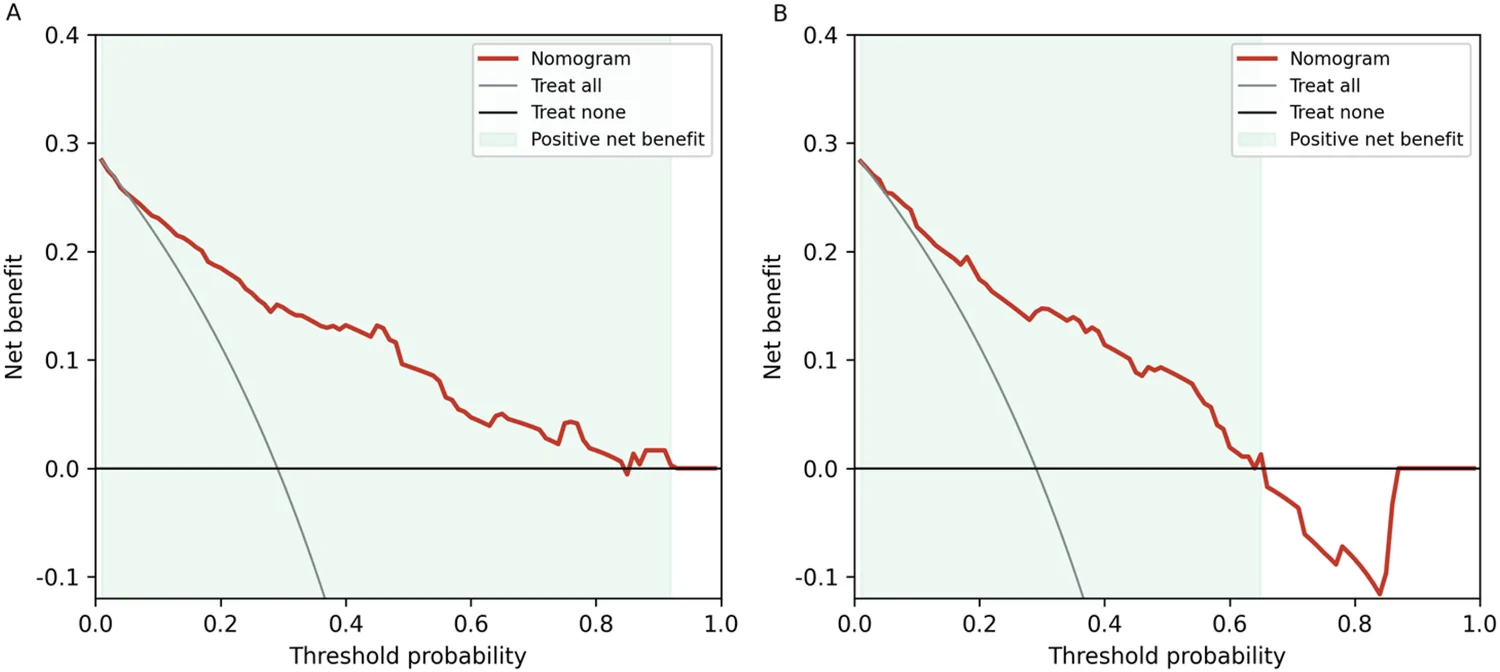
Decision curve analysis of the frailty screening model. **(A)** Training set. **(B)** Validation set.

**Figure 6:**
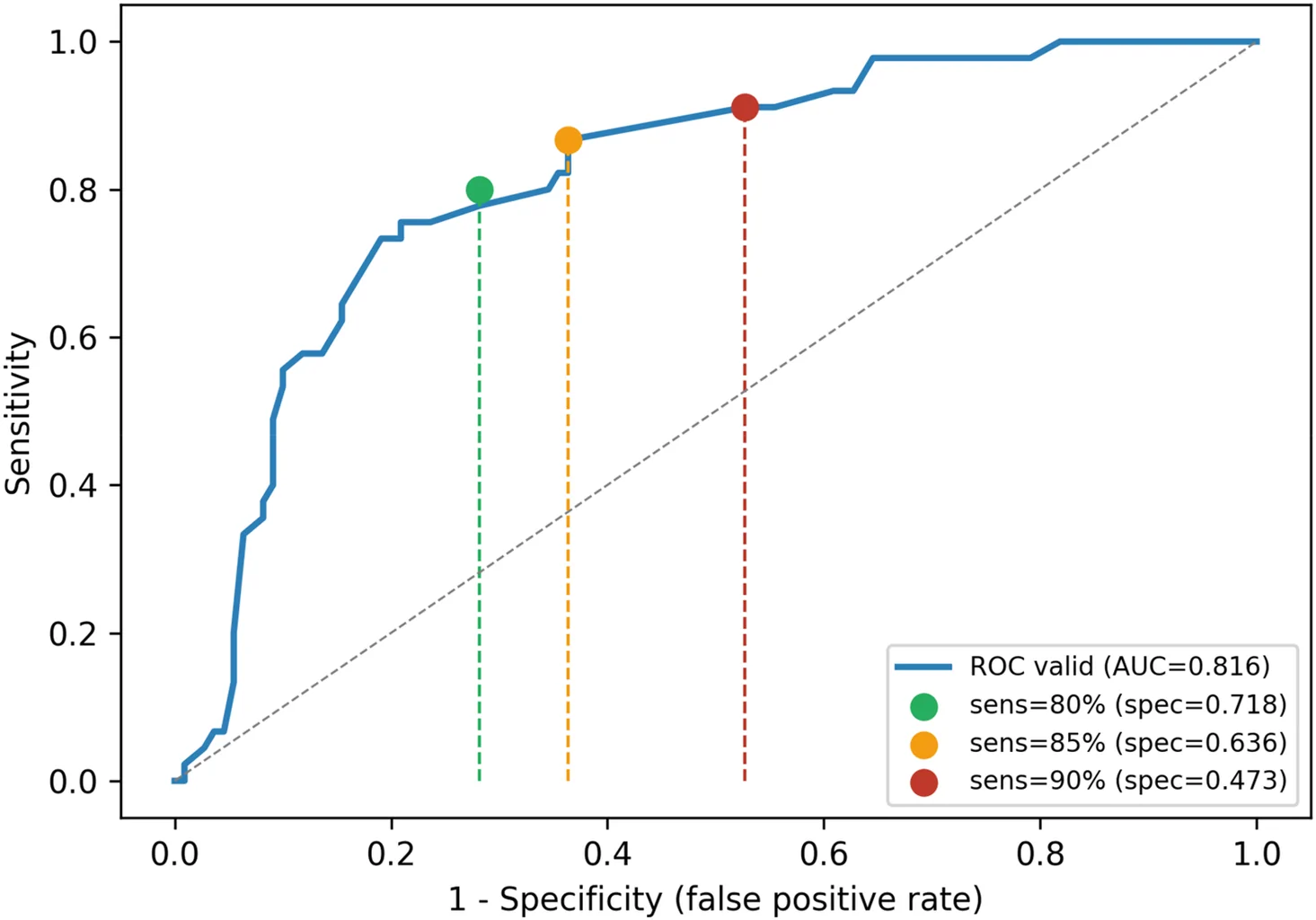
Operating-point sensitivity at targeted sensitivities (validation set). The ROC curve of the validation set is shown with the operating points corresponding to sensitivity targets of 80%, 85%, and 90%.

### 3.5 Sensitivity, robustness, and supplementary analyses

Several sensitivity, robustness, and supplementary analyses were performed on the training and validation sets with the same 7:3 stratified split (seed = 345) to examine study positioning, model stability, calibration, and clinical utility. The detailed results of these analyses are summarized in Supplementary Table S2 of the Supplementary Materials.

The key findings are as follows. LASSO regression confirmed the stability of the five retained predictors (Supplementary Table S2, item A). Ordinal logistic regression supported the preservation of all five associations across the non-frail/pre-frail/frail gradient, and the proportional-odds assumption was not rejected (*P* > 0.05; Supplementary Table S2, item B). Restricted-cubic-spline analysis supported categorical age modelling over non-linear splines (Supplementary Table S2, item C).

## 4 Discussion

This study found that the prevalence of frailty (FRAIL scale score ≥ 3) among older adults with cataract was 29.07% in the present sample, with an additional 33.33% classified as pre-frail and 37.60% as non-frail. Although the present estimate is lower than the 58.88% reported in a single-centre Chinese study using the same FRAIL-based definition (13), possibly reflecting differences in case-mix or in the underlying frail population, it remains substantially higher than the 10% pooled estimate for community-dwelling Chinese older adults (6) and the 15.8% reported in cognitively unimpaired older adults (25). The high prevalence observed in this study highlights the importance of identifying frailty in elderly cataract patients, while recognising that the present cross-sectional design describes associations at a single time point. The model should therefore be positioned as a tool to identify concurrent frailty status in this outpatient population.

Advanced age was a factor independently associated with frailty in the present multivariable model, consistent with previous studies (26, 27). The age category ≥ 80 years showed a substantially higher odds ratio than the 70–79 years category, and the likelihood-ratio comparison of the categorical age specification with a continuous age term favoured the categorical specification (chi-square = 6.32, df = 1, *P* = 0.012), suggesting that the relationship between age and frailty in this sample is not strictly linear. IADL serve as a key indicator of an individual's integrated physiological function, cognitive ability, and interaction with the environment. Impairment in IADL is associated with — and may precede — declines in basic activities of daily living and is regarded as a sensitive marker of cognitive decline and frailty in older adults (28, 29). In the present dataset, the Lawton IADL Scale includes activities such as shopping, transportation, telephone use, housekeeping, and medication management, several of which are closely related to functional independence. Because functional dependence is itself one of the defining domains of frailty, some overlap between the IADL items and the frailty outcome is intrinsic to this type of model, and the IADL association should not be interpreted as independent of functional limitation in general. The present Discussion therefore distinguishes between associations observed in the cross-sectional data and the previously reported mechanisms — such as activity restriction, muscle atrophy, and reduced cognitive stimulation — that have been hypothesised to link IADL impairment to the development of frailty (30, 31). Frailty status is itself associated with poorer quality of life (32), but the present design cannot determine the direction of that association.

Previous cross-sectional and longitudinal studies have reported an association between visual impairment and depressive symptoms (33, 34), and a bidirectional relationship between depression and frailty has been described in Mendelian-randomization and neuroimaging studies (35, 36). In the present cross-sectional data, depression was independently associated with frailty; the direction of this association cannot be inferred from the present design. Self-rated health status, as an individual's subjective perception of their overall health, often captures health information that may not be easily quantified by clinical examinations (37); poor self-rated health is consistently associated with a heavier disease burden and with adverse outcomes in older adults. In the present data, the poor-self-rated-health  ×  frail stratum contained a limited number of events, which is reflected in a wide confidence interval (OR 19.03, 95% CI 5.82–62.22); the point estimate should be interpreted with caution. In addition, cataract patients face a higher fall risk than the general population (38), and previous literature has reported an association between a history of falls and subsequent functional decline (39–41); the present data confirm only the cross-sectional association between a history of falls and concurrent frailty status.

Because the FRAIL scale is a brief, well-validated frailty screen (16, 17), a natural question is whether a multi-item model adds value. We emphasise that the present model was not developed to replace the FRAIL scale: the FRAIL scale itself defined the outcome in this study, so a direct claim of superiority over it would be circular, and such a comparison must await an independent cohort with a separate frailty reference standard. The contribution of the present model lies instead in how it can be deployed. In a busy ophthalmology outpatient clinic, consultation time is short, and eye-care staff rarely have the time to administer a dedicated frailty instrument — including grip-strength measurement and structured enquiry about fatigue and weight loss — to every older patient. All five predictors retained here (age, IADL, self-rated health, depression, and history of falls) are items already captured in the routine electronic record or in the standard geriatric history taken during cataract assessment. The model can therefore be computed automatically from information that is collected in any case, providing opportunistic, zero-burden screening. In this role it functions as a triage step: it stratifies the outpatient population and flags the subset at highest probability of concurrent frailty for referral to comprehensive geriatric assessment, rather than attempting to substitute for a formal frailty instrument. The continuous probability and point-based visual score may additionally aid clinician–patient communication and shared decision-making. Whether this zero-burden strategy identifies as many frail patients as a dedicated FRAIL assessment, and at what cost, remains an empirical question for a future head-to-head study.

As discussed above, the presence of frailty in older adults with cataract is associated with multiple factors. Integrating these factors into a comprehensive assessment may help clinicians identify individuals with concurrent frailty, so that appropriate geriatric or multidisciplinary evaluations can be considered. We emphasise that the present cross-sectional design supports screening for current frailty status, not prediction of incident frailty.

Taken together, the five retained factors discriminated concurrent frailty better than demographic and comorbidity information alone, and the calibration analyses showed that the predicted probabilities were conservative rather than over-confident. For a triage application this is the safer direction of error, because an under-estimated probability would be corrected by the confirmatory geriatric assessment that follows a positive screen. The detailed performance estimates are reported in Section 3.4.

This study has several limitations. First, the cross-sectional design and convenience sampling from a single tertiary hospital preclude causal inference and prediction of future events such as falls, fractures, or healthcare utilisation. Second, we did not collect cataract-specific clinical variables — objective visual acuity, cataract type, cataract severity, or laterality — so the final predictors are predominantly general geriatric factors rather than disease-specific indicators; because the available ophthalmic information was limited to self-report, the association between IADL impairment and frailty may partly reflect vision-related restriction of daily activities. Third, the sensitivity at the conventional Youden cutoff (57.8%) is moderate, and higher sensitivity can only be achieved at the cost of reduced specificity and positive predictive value; the threshold should therefore be selected deliberately according to the intended use. Fourth, the calibration slope exceeded 1, indicating that the spread of the predicted probabilities was conservative relative to the observed event proportions; external recalibration (intercept and slope adjustment) would further refine probability estimation in more diverse populations. Fifth, the outcome was defined by the FRAIL scale, which itself served as the reference instrument; a head-to-head comparison against an independent frailty reference standard in an external cohort is required before any incremental value over the FRAIL scale can be claimed. Future multi-centre studies with external validation, collection of cataract-specific clinical variables, and external recalibration are needed to support the generalisability of the model.

## 5 Conclusion

Frailty is common among older adults attending cataract outpatient clinics, and the strong yet modifiable nature of its associated factors—functional dependence, poor self-rated health, depression, and a history of falls—suggests a clear window for intervention at the point of routine eye care. By repurposing information that clinicians already collect, the present model converts an ordinary cataract visit into an opportunity to flag concurrent frailty and to route the most vulnerable patients toward comprehensive geriatric assessment, without adding any measurement burden. In doing so, it reframes frailty detection in ophthalmology from a reactive, symptom-driven task into a proactive, integrated step in the continuum of care. Because the model is built and validated within a single centre and the FRAIL scale itself defined the outcome, its value should be regarded as generative rather than definitive: it demonstrates that opportunistic screening in this setting is both feasible and informative, while the work of confirming its performance, its incremental benefit over the FRAIL scale, and its generalisability across diverse ophthalmic and geriatric populations remains. If validated externally, this approach could support a low-cost pathway through which eye-care services contribute meaningfully to the earlier recognition and holistic management of frailty in an ageing population.

## Data Availability

The original contributions presented in the study are included in the article/Supplementary Material, further inquiries can be directed to the corresponding author.
