# Supplementary material for: Construction and validation of a frailty screening model for older adults with cataract: a cross-sectional study
Source: Front Med (Lausanne). 2026 Oct 1;13:1934999. doi: 10.3389/fmed.2026.1934999 (PMC13668520; doi:10.3389/fmed.2026.1934999)
Supplement: Supplementary file 1 [file Datasheet1.docx]

**Supplementary Materials**

Manuscript title: "Construction and validation of a frailty screening model for older adults with cataract: a cross-sectional study"

Journal: Frontiers in Medicine (Geriatric Medicine)

*Contents: Supplementary Table S1; Supplementary Table S2*

**Supplementary Table S1. Training-set univariate logistic regression of frailty among older cataract outpatients (n = 361).**

| **Variable** | **Level** | **N (training)** | **OR (95% CI)** | **P** |
| --- | --- | --- | --- | --- |
| Gender | Male (ref) | 136 | 1.00 (ref) | 0.559 |
|  | Female | 225 | 0.870 (0.546–1.387) | 0.559 |
| Age | 60-69 (ref) | 100 | 1.00 (ref) | 0.001 |
|  | 70-79 | 172 | 2.001 (1.080–3.708) | 0.028 |
|  | ≥80 | 89 | 3.638 (1.862–7.108) | <0.001 |
| Educational level | Primary school or below (ref) | 258 | 1.00 (ref) | 0.830 |
|  | Junior high school | 57 | 1.126 (0.606–2.093) | 0.707 |
|  | Senior high school or above | 46 | 0.861 (0.423–1.753) | 0.680 |
| Marital status | Married (ref) | 251 | 1.00 (ref) | 0.044 |
|  | Unmarried | 110 | 1.635 (1.012–2.643) | 0.045 |
| Residence type | Urban (ref) | 205 | 1.00 (ref) | 0.279 |
|  | Rural | 156 | 1.286 (0.815–2.031) | 0.280 |
| Living arrangement | Living with others (ref) | 332 | 1.00 (ref) | 0.810 |
|  | Alone | 29 | 1.106 (0.486–2.516) | 0.810 |
| Medical insurance | Yes (ref) | 354 | 1.00 (ref) | 1.000 |
|  | No | 7 | 0.975 (0.186–5.105) | 0.976 |
| Per capita monthly household income (yuan) | ≥2000 (ref) | 143 | 1.00 (ref) | 0.706 |
|  | <2000 | 218 | 1.094 (0.686–1.744) | 0.706 |
| Comorbid chronic diseases | No (ref) | 90 | 1.00 (ref) | 0.003 |
|  | Yes | 271 | 2.486 (1.352–4.572) | 0.003 |
| Alcohol consumption | Non-drinker (ref) | 329 | 1.00 (ref) | 0.030 |
|  | Drinker | 32 | 0.322 (0.110–0.944) | 0.039 |
| Smoking status | Non-smoker (ref) | 316 | 1.00 (ref) | 0.703 |
|  | Smoker | 45 | 0.872 (0.431–1.762) | 0.703 |
| Annual physical examination | Yes (ref) | 228 | 1.00 (ref) | 0.013 |
|  | No | 133 | 1.792 (1.127–2.849) | 0.014 |
| Social activity participation | Yes (ref) | 154 | 1.00 (ref) | 0.003 |
|  | No | 207 | 2.077 (1.282–3.365) | 0.003 |
| Physical exercise | Yes (ref) | 281 | 1.00 (ref) | <0.001 |
|  | No | 80 | 3.825 (2.272–6.438) | <0.001 |
| Social support | Yes (ref) | 322 | 1.00 (ref) | 0.212 |
|  | No | 39 | 0.599 (0.266–1.350) | 0.216 |
| Hearing status | No (ref) | 280 | 1.00 (ref) | 0.217 |
|  | Yes | 81 | 1.393 (0.822–2.361) | 0.219 |
| IADL impairment | No (ref) | 108 | 1.00 (ref) | <0.001 |
|  | Yes | 253 | 7.772 (3.622–16.677) | <0.001 |
| Self-rated health status | Good (ref) | 61 | 1.00 (ref) | <0.001 |
|  | Fair | 220 | 4.300 (1.488–12.425) | 0.007 |
|  | Poor | 80 | 23.750 (7.825–72.085) | <0.001 |
| Depression | Normal (ref) | 250 | 1.00 (ref) | <0.001 |
|  | Mild depression | 93 | 4.227 (2.524–7.080) | <0.001 |
|  | Severe depression | 18 | 8.638 (3.084–24.198) | <0.001 |
| Anxiety | Normal (ref) | 293 | 1.00 (ref) | 0.016 |
|  | Mild anxiety | 53 | 1.424 (0.763–2.660) | 0.267 |
|  | Moderate anxiety | 14 | 4.985 (1.621–15.330) | 0.005 |
|  | Severe anxiety (not estimable: separation) | 1 |  |  |
| Sleep quality | Very good (ref) | 69 | 1.00 (ref) | <0.001 |
|  | Okay | 173 | 1.798 (0.867–3.731) | 0.115 |
|  | Fair | 112 | 3.541 (1.678–7.475) | 0.001 |
|  | Very poor | 7 | 13.182 (2.263–76.771) | 0.004 |
| History of falls | No (ref) | 276 | 1.00 (ref) | <0.001 |
|  | Yes | 85 | 2.698 (1.622–4.485) | <0.001 |

*OR, odds ratio; CI, confidence interval; ref, reference category. Variables with P < 0.05 in the training set entered the multivariable model (see Methods, Section 2.3).*

**Supplementary Table S2. Summary of sensitivity, robustness, and supplementary analyses (all performed on the training and validation sets with the same 7:3 stratified split, seed = 345).**

| **Analysis** | **Outcome measure** | **Result (validation set unless noted)** |
| --- | --- | --- |
| (A) LASSO on 22 prespecified predictors | Validation AUC; retained predictors | 0.812 (0.741-0.882); 17 of 29 non-zero coefficients retained (all 5 final predictors in the reduced model) |
| (B) Three-level ordinal logistic (non-frail / pre-frail / frail) | Ordinal ORs and proportional-odds test | All 5 associations preserved; PO assumption not rejected (LR test P>0.05) |
| (C) Age specification (categorical vs continuous vs RCS) | Validation AUC; LR test | Categorical 0.816; Continuous 0.781; RCS 0.773; categorical > continuous (P=0.012); RCS not better than linear (P=0.754) |
| (D) Post-hoc sample-size check | Minimum N for 5-10 predictors at observed 29.07% rate | 246 (5 EPV) - 492 (10 EPV); achieved N=516 above both |
| (E) Separation: collapse severe-anxiety cell | Validation AUC | 0.816 (unchanged); severe anxiety cell auto-dropped (1/361) |
| (F) Stronger baseline (age+sex+comorbid) | Validation AUC; DeLong; NRI; IDI; Brier | 0.708; DeLong P=0.026; NRI=0.383; IDI=0.1897; Brier 0.1858 -> 0.1553 |
| (G) Bootstrap with full model-development procedure (5000 resamples) | Optimism-corrected C; 95% CI | 0.810; 95% CI 0.780-0.841 (mean optimism 0.031; mean n_finalvars 5.4) |
| (H) DCA positive-net-benefit range | Threshold range | Training 0.01-0.92; validation 0.01-0.65 |
| (I) Threshold sensitivity at 80/85/90% | Specificity, PPV, NPV at each target | 80%: spec 0.718, PPV 0.537, NPV 0.898; 85%: 0.636, 0.494, 0.921; 90%: 0.473, 0.414, 0.929 |
| (J) Subset aged >=65 years | Validation AUC; final predictors | 0.745 (0.655-0.836); 2 predictors (SRH, Falls) retained |
| (K) Internal vs external validation terminology | Terminology change | 70/30 described as held-out INTERNAL validation throughout; external validation explicitly listed as future work |
| (L) Calibration intercept, slope, Brier (reported in Section 3.4) | Training; validation | Intercept: -2.80; -2.54. Slope: 5.64; 4.73. Brier: 0.1395; 0.1553 |

*AUC, area under the ROC curve; LASSO, least absolute shrinkage and selection operator; RCS, restricted cubic splines; EPV, events per variable; NRI, net reclassification improvement; IDI, integrated discrimination improvement; DCA, decision curve analysis; PPV/NPV, positive/negative predictive value; SRH, self-rated health; PO, proportional odds.*
